# Prognosis and Follow-Up of Idiopathic Pulmonary Fibrosis

**DOI:** 10.3390/medsci6020051

**Published:** 2018-06-14

**Authors:** Estrella Fernández Fabrellas, Ricardo Peris Sánchez, Cristina Sabater Abad, Gustavo Juan Samper

**Affiliations:** 1 Unit of Interstitial Lung Diseases, Pneumology Service, Hospital General Universitario, 46014 Valencia, Spain; sabaterabadcristina@gmail.com (C.S.A.); gustavo.juan@uv.es (G.J.S.); 2 Hospital Sant Francecs de Borja, Gandía, 46702 Valencia, Spain; ritxiperis@gmail.com

**Keywords:** idiopathic pulmonary fibrosis, prognostic factors, monitoring, follow-up

## Abstract

Idiopathic pulmonary fibrosis (IPF), a devastating progressive interstitial lung disease (ILD) with no known cause, is the most common and deadly of the idiopathic interstitial pneumonias. With a median survival of 3–5 years following diagnosis, IPF is characterized by a progressive decline in lung function and quality of life in most patients. Prognostic factors recognized classically that influence mortality include functional, clinical and radiological parameters. However, in recent years, there has also been progress in the knowledge of genetic factors and biomarkers that may be useful in the prognostic evaluation of these patients. On the other hand, the monitoring of the disease throughout its evolution is key to improving the prognosis of the patients, as it allows for taking therapeutic measures based on this evolution, even early remission for lung transplantation. This article reviews the main prognostic factors of the disease, as well as the most useful way to monitor the disease follow-up.

## 1. Introduction

Evaluation of Idiopathic Pulmonary Fibrosis (IPF) prognosis is based on the determination of disease progression, stability or improvement, using not only the most important ones, lung function parameters, but also others such as clinical, radiological and even serological. Idiopathic pulmonary fibrosis is characterized by pulmonary function tests (PFT) disorders with a typical restrictive pattern defined by a decreased total lung capacity (TLC) or vital capacity (VC) altogether with the decrease of the diffusion of carbon monoxide (DLco), including corrections by alveolar volume (KCO). The values of these parameters and also some patient characteristics can help to classify the initial severity of the disease at the time of diagnosis and this could confer a baseline prognosis. However, these PFT absolute values at diagnosis are not still as important as how much they change in time defining the speed of disease progression. In other words, these changes in time are better for determining the stability or progression of the disease so obviously will determine IPF prognosis (Table 1).

This article is focusing on a detailed analysis of clinical and functional characteristics, and also which updated explorations have shown to have a prognostic value and are expected to be fundamental to the proper approach of the patient progression.

## 2. Clinical Parameters

The clinical conditions that have been associated with a worse prognosis are diverse and depend on epidemiological data such as male gender and age, symptoms such as baseline dyspnoea, or characteristics of the disease evolution expecting a phenotype or another depending on the exacerbations. In addition, the presence of comorbidities and complications such as emphysema, pulmonary hypertension, cardiovascular diseases and bronchogenic carcinoma are clinical factors that also have been associated with more rapid progression of IPF. Therefore, a rapid progression of IPF is more common in males with a personal smoking history and other co-morbidities, altogether with a delay in diagnosis. Thus, the delay in the evaluation by a team of experts in IPF may be related to a shorter survival, regardless of the level of severity or other prognostic factors [1]. In the same way, it is known that not all patients with IPF evolve in the same way. This is why different phenotypes of IPF are now being highlighted, with two distinct groups—on the one hand, those whose evolution is slowly progressive, with interspersed exacerbation episodes that can be a clear decline in the survival of the patient [2], and, on the other hand, those who suffer exacerbations frequently, encouraging to a clinical and functional faster decline and consequently to an earlier mortality [3]. This situation is concerning because there are no predicting factors strong enough to identify who will evolve in one way or another. The explorations to be performed in the follow-up and patient monitoring will differ depending on the clinical status of the patient at the time of diagnosis. In those cases with very little functional impact and absence of limitation to the exercise assessed by the six minutes walking test (6 MWT), evaluation of the patient every three or six months should include the degree and intensity of cough using the questionnaire of Leicester and dyspnoea, using valid scales such as the modified Medical Research Council (MRC), Borg, and both initial and transition dyspnoea indexes. In cases of rapid and progressive disease, the visits should be more frequent (every three months or less, according to evolution), and should also review the cough, the degree of dyspnoea and the progression of the functional limitation by PFT, including arterial blood gases and the 6 MWT [2]. The plain chest X-ray can provide a lot of information showing complications that could be influencing the clinical and or functional worsening of the patient. Registering hospitalizations related to any respiratory cause (exacerbations, infections, etc.) is very important because of their close association with an increase in mortality during the follow-up.

## 3. Comorbidities

The presence of pulmonary hypertension at the diagnosis or follow-up is a factor of bad prognosis related to a worsening in the survival of patients with IPF [4]. Echocardiography is a non-invasive technique that allows for evaluating the existence of pulmonary hypertension without any risk and is strongly recommended in patients with both IPF and emphysema at the diagnosis, and annually in the follow-up. It is also mandatory doing the ecocardiography in patients with the “frequent exacerbator” phenotype and in patients with lung transplant criteria. The right heart catheterization is exclusively indicated in patients with definitive diagnosis of IPF in the following situations: (1) before the lung transplantation; (2) in cases of clinical deterioration, limitation of the ability to exercise, drop in the DLco (especially if DLco < 40% of the predicted) or disproportionate hypoxemia with the restrictive ventilatory decline, especially if there is also emphysema; (3) if it is considered essential to a strict evaluation of the prognosis; (4) if serious precapillary pulmonary hypertension diagnosed in echocardiography (flow from tricuspid regurgitation > 3.5 m/s) is suspected to evaluate the possibility of administering treatment for pulmonary hypertension; and (5) if left ventricular dysfunction with preserved systolic function is suspected [5,6]. Emphysema has been proposed as a factor of bad prognosis at the time of diagnosis of IPF, otherwise it apparently seems that is not only explained by the worsening of the interstitial disease itself, but also because of its additive effect in terms of pulmonary emphysema disorder [7]. It is likely that a more severe decline in the levels of DLco added to the effect of pulmonary arterial hypertension as a complication of both disorders could be responsible for the worsening of prognosis [8].

## 4. Functional Parameters

Progression, stability or improvement of the disease are classically defined by lung function criteria [9]. Functional parameters used in the monitoring and evaluation of the disease are the percentage value on the predicted forced vital capacity (FVC) and DLco, the pulse-oximetry at rest and 6 MWT data. Among them, the decrease in FVC % is the parameter of lung function that best predicts mortality. Changes between 6 or 12 months in the percentage of FVC or DLco define the worsening, stability or improvement of the disease [10]: a decline greater than or equal to 10% of the FVC or greater than or equal to 15% of the DLco are considered the cut-off points to define disease progression and therefore are poor prognosis factors. However, although these variations have been postulated as the best predictors of mortality, some authors have shown that some patients have better survival than expected despite losses greater than 10%, while others have a worse prognosis despite maintaining stable FVC values in time [9].

As the values of lung function parameters in the early stages of the disease may be practically normal, the hypothesis that annual variability could be lower is quite possible with less accused FVC changes meaning also worsening or progression of the disease. The decline between 5% and 10% of FVC or DLco as the significant cut-off point is currently accepted to be worsening, and, therefore, able to identify patients with worse prognosis and shorter survival. Some authors have shown that the decline in FVC > 5% (from its absolute value) and >10% (from its absolute or relative value) within the 6–12 months follow-up indicate a worse prognosis [2]. On the other hand, it is estimated that the minimal difference clinically relevant of the FVC decline is from 2 to 6%, although we must keep in mind that, in patients with IPF-emphysema, lung volumes could be normal or very little affected, so this decrease of the FVC does not predict survival, more specifically, if emphysema has an extension of more than 15%, then it is proposed that the utility of the FVC in the evaluation of the IPF is worthless [11]. The six-monthly or annual fall >10% of FVC or >15% of DLco, which are considered as the gold standard to establish the prognosis of the disease, are no longer providing a real and complete evaluation of the patient’s risk. The FVC has been shown superior to DLco to establish the prognosis of the disease taking into account their changes in time; however, the DLco can be considered the main not temporary parameter to evaluate prognosis so the determination of DLco less than 35–40% of the predicted seems to be related to less than two years of survival [2,12].

Six-min walking test (6 MWT): The tests of maximum effort are other accepted tools to determine the initial severity of IPF, and also to assess its evolution and to determine the prognosis [13]. Among the exercise tests, 6 MWT is currently the most used because its excellent assessment of the prognosis, though which of the measures obtained during the test would be the best parameter to assess the IPF progression is not yet clearly defined [14]. The 6 MWT has generally been used in heart diseases, chronic obstructive pulmonary diseases (COPD) and pulmonary hypertension [15,16], and its usefulness in these diseases is mainly based on the difference between the oxygen saturation by pulse oximetry (SpO_2_) level at the beginning and at the end of the walk, i.e., the total desaturation, as well as the decrease of the distance walked at successive records. The parameters of the 6 MWT considered as predictors of worse survival in IPF are also the SpO_2_ measurement at the end of the six minutes and the distance walked. Thus, the SpO_2_ below 88% is an indicator of poor prognosis [17,18]. In addition, desaturation level reached at the end of the test can discriminate which of the functional parameters will be most appropriate to evaluate the follow-up of the patient, i.e., if SpO_2_ is ≤88%, a decline >15% of the DLco in six months is the best predictor mortality, and if SpO_2_ > 88%, the most appropriate parameter to evaluate progression will be the decline >10% of the FVC. Considering the distance walked in the test (6 MWD), the risk of mortality the first year is two times greater if it is less than 250 m, and three times greater if the distance walked after 24 weeks decreases 50 m or more. The minimal clinically important difference (MCID) is set at a distance of 24–45 m [17,18].

## 5. High-Resolution Computed Tomography

The importance of the High-Resolution Computed Tomography (HRCT) in IPF is not only for its value as a diagnostic tool strong enough to relegate to a second step the surgical open lung biopsy, which was considered until just a few years ago as the gold standard for diagnosis [19,20,21], but also for its relevant role in monitoring and evaluating prognosis of the disease, considering that HRCT is able to determine the initial extension and its progress over time. Because of this role in the monitoring of the disease, the HRCT should not only describe and locate IPF findings, but also quantify the extent of those findings, which certainly is essential to assess the progression of the disease beyond the functional or clinical data. Similarly, in more advanced stages of the disease, clinical and functional deterioration of the patient can make it impossible to determine the severity tested by functional parameters, so the HRCT would be the alternative tool that could determine objectively the progression of the IPF quantifying the extent and progression of fibrosis. Some studies have shown a good correlation between the PFT and the extension of IPF in HRCT findings [22,23], identifying factors of worse prognosis or early mortality using quantified parameters, especially focusing on the extent of reticulation, honeycombing and traction bronchiectasis. Although several quantifying methods have been proposed, i.e., quantitative or automated reading and semi-quantitative visually read by radiologists, in order to measure the extent of the variated findings of IPF in the HRCT [19,23], and, while all international guidelines recommend quantifying those findings, none of these methods has been postulated for routine use, nor at the time of the diagnosis nor in the follow-up of patients. Although most quantitative analyses collected in the literature did not improve the visual or semi-quantitative methods, a new system of automatic analysis called CALIPER (Computer-Aided Lung Informatics for Pathology Evaluation and Rating) is capable of measuring new alterations, such as the size of the vessels pulmonary (PVV), is highly-related mortality in IPF patients [24]. In any case, it is not recommended to perform HRCT as a matter of routine in the follow-up of patients with IPF except to confirm the clinical suspicion of specific situations, such as the progression of the disease, the suspicion of acute exacerbation or the presence of complications or other comorbidities, principally pulmonary neoplasia, pulmonary embolism, emphysema or pulmonary hypertension.

## 6. Biomarkers

Despite there not being a single biomarker in widespread clinical use to establish the prognosis or to use during the monitoring of the IPF, recent studies with some serum markers are found to be related to the prognosis of the disease (Table 2).

Many biomarkers have been evaluated according to physiopathological complex ways of fibrosis such as epithelial damage indicators Krebs von den Lungen-6 (KL-6), mucin 1 (MUC-1) and surfactant protein A and D (SP-A and SP-D) and other markers of activation of alveolar macrophages (chemokine (C-C motif) ligand 18, CCL-18), neutrophils (S100A12, interleukin-8 (IL-8)), markers of stress oxidative intercellular adhesion molecule 1 (ICAM-1) and vascular cell adhesion molecule 1 (VCAM-1) [25]. Some cytokines, i.e., CCL18 and chemokine (C-X-C motif) ligand 13 (CXCL13), are influential in the migration of B-lymphocytes and macrophages activation, and thus have been associated with increased mortality [26,27]. Matrix metalloproteinase 7 (MMP-7) has been proved to be a useful marker for the decline of the FVC and also for the progression of the disease because the increase in its value with the time, or simply staying high in the blood levels, determines a worse prognosis in patients with IPF [28]. The increase of the matrix metalloproteinase 9 (MMP-9) in the bronchoalveolar lavage (BAL) has been associated with rapid progression of the disease [3]. On the other hand, there is a protein located at the epithelial tissue and is part of the alveolar repair mechanism, the integrin protein αvβ6, which has been associated with an increase in mortality. It is possible to detect it with a lung biopsy [29]. Finding lots of fibroblast foci in different sites of surgical lung biopsy and detection of fibrocytes in peripheral blood could interact with lower survival. Finally, the increase in serum of LOXL2 (lysyl oxidase-like-2) seems to relate also with worse prognosis, but a reliable validation is still required. A recent study associated with the pulmonary microbiome in BAL with the evolution of IPF, suggesting that the decrease in the diversity of the present microorganisms (Bacteroides, Fimicutes, Proteobacteria) would determine a worst prognosis due to their correlation with levels of FVC, values of the 6 MWT, and even with the SP-D levels [30]. Genetics have been also studied and could also have a role in IPF prognosis. *MUC5B* gene polymorphism has been proposed as a marker of better prognosis in several clinical trials. In addition, together with shortening of telomeres could configure special characteristics in asymptomatic patients, which would mean a higher risk of developing IPF [31]. Shortening of telomeres influences the epithelial regeneration, and its lower length in white blood cells has been associated with a worse prognosis of the IPF [32]. The determination of molecular biomarkers could lead in the near future to the management of the IPF with prognostic endotypes and also diagnostic and therapeutic ones [33].

## 7. Multidimensional Scales

In recent years, multidimensional indices have been developed to identify the most accurate prognostic factors in IPF, and also to establish a practical and clinically useful method to integrate them and predict individual mortality risk.

As occurs in other chronic respiratory diseases, multidimensional scales promote the modification of disease prognostic assessment moving from unifactorial and compartmentalized evaluation (in IPF by PFT, 6 MWT or HRCT) to this new concept of risk transversality.

The first study in this regard conducted by Du Bois [34] with data from two clinical trials using interferon gamma has established a clinical model composed of four factors (age, hospitalization, % baseline FVC and change in FVC in 24 weeks) for mortality risk prediction at one year (Table 3).

Recently, these authors have reported an improvement in this scale mortality prediction by adding two more factors: 6 MWD travelled at baseline and at 24 weeks [15].

From retrospective data analysis of three large geographically different patient cohorts from the USA and northern Italy, the GAP Index (Gender, Age, Physiology) arises, which includes four baseline variables: sex, age and two functional variables, % FVC and % DLco. Using this GAP index, the authors define stages I, II and III of individual mortality estimated at one, two and three years [35] (Table 4).

A modified GAP index has included radiological variables instead of % DLco and it allows a more integrated prognostic evaluation by taking into account the extent of fibrosis in HRCT, and also evaluating reticulation and honeycombing patterns through a quantitative scoring system.

The original GAP is a validated model for mortality risk prediction at diagnosis, however it does not include patient’s evolutive variables, which are known to contribute to risk prediction in IPF. The use of these evolutionary variables can contribute to improve GAP predictive performance by providing a longitudinal assessment of this risk. Thus, the so-called Longitudinal GAP [36] arises including original GAP baseline variables plus hospitalizations due to respiratory causes and changes in % FVC at 24 weeks (Table 5).

The use of GAP at the time of diagnosis along with the longitudinal GAP in patient follow-up is considered to be very useful to unify mortality risk prediction in IPF.

The Risk stratificatiOn ScorE (ROSE) arises from prospective analysis of 138 patients and jointly assesses the degree of dyspnoea measured by the modified MRC scale, 6 MWD data, and Composite Physiological Index (CPI), which includes FVC, FEV1 and DLco. The Risk stratificatiOn ScorE (ROSE) allows patients to be classified on three risk levels (low, intermediate or high), establishing 3-year mortality in 19%, 42% and 100%, respectively, for each level [37] (Table 6).

So far, the data we know has not shown an optimal correlation between PFTs, dyspnoea, exercise capacity, and extension of IPF in HRCT, so it is not surprising that pulmonary function data by themselves are insufficient to stratify the disease. Therefore, this could be a serious limitation when planning further studies in IPF in order to ensure effective treatment options. The recently published algorithm CALIPER has shown better correlation with respiratory functional test variables than semi-quantitative visual analysis of scan images. The authors have also shown that stratification using CALIPER and CPI variables predicts mortality more strongly than using GAP index alone. Specifically, a CALIPER variable, the pulmonary vessel volume (PVV), is the one that is more robustly related to mortality, so, in the future, it could be a new index in IPF evaluation [24].

Multidimensional indices proposed at this moment have been shown to improve the predictive power in the expected evolution of patients with IPF. Due precisely to their multidimensional nature, these scales are able to integrate diverse domains of disease pathophysiology and provide a wider prognostic information. However, the optimal multidimensional prognostic index for IPF is yet to be defined. All indices published up to now have several limitations in design, methodology, type of population, follow-up duration or patient’s number. Those indices derived from prospective clinical trials generally include patients with mild or moderate disease and may underestimate the real prevalence of exacerbations, which are frequent in advanced IPF. On the other hand, those derived from retrospective studies tend not to include patients with rapidly progressive IPF, thus underestimating current mortality rate and are deficient in the assessment of each functional variable modification during follow-up, so these important data often cannot be conveniently valued for losses during the follow-up period.

These multidimensional scales have shown their usefulness in predicting mortality in IPF. However, they do not predict significantly the trajectory of lung function decline. In two recent studies, one conducted by Salisbury et al. [38] and another by Ley et al. [39] and, in agreement with what was previously published in the literature, lung function progression does not vary according to disease severity defined by the GAP index nor by clinical variables. This is probably due to the heterogeneous nature of the disease. For this reason, studies that identify predictors of rapid or slow lung function decline are necessary, which would undoubtedly help to establish a more accurate prognosis and more adequate treatment. As both authors point out, it is probable that these factors have a biological nature (molecular and/or genetic) and are not related to demographic characteristics nor radiological images or pulmonary physiology.

The optimal multidimensional index should probably emerge from a multicenter effort as a prospective study involving a large number of new patients, a reasonable number of variables, with a sufficiently long follow-up period in order to reflect adverse events and mortality. It also should implement a systematic and longitudinal follow-up of those variables considered fundamental for the analysis. In addition, it should be simple to calculate and it should include safe, cheap and easily measurable variables.

Undoubtedly, a multidimensional index with sufficient predictive power for survival could represent the main practical, sensitive and specific objective for future clinical trials in IPF, thus contributing to the development of new therapeutic options for this devastating disease.

Currently, there is not enough scientific evidence to reliably support any of these scales, so, to date, no current clinical guideline has included or recommended their systematic use in the management of IPF. In the same way, it still remains to determine its guiding role for therapeutic decisions.

## 8. Follow-Up

The main reasons for monitoring patients with Idiopathic Pulmonary Fibrosis are to evaluate the disease progression that can lead to a change in treatment and identify comorbidities and complications that may influence when to initiate or stop antifibrotic treatment, when to establish oxygen therapy, when to refer to lung transplantation and when to initiate palliative measures.

Currently, there is no consensus on how to monitor these patients. Most recommendations are based on expert opinion and extrapolated evidence. The follow-up strategy is based on clinical need assessed on a case-by-case basis. The frequency and type of follow-up varies with the disease course with symptom assessment and pulmonary function measurements being the cornerstones of disease monitoring.

In early disease stages, patient follow-up is usually done at three- to six-month intervals. However, in patients with more advanced or rapidly progressive disease, a closer monitoring is performed every three months or less [13] (Table 7).

### 8.1. Evaluation of Symptoms

The most frequent symptoms in IPF are dyspnoea, reduced exertional capacity and unproductive or minimally productive cough. These symptoms are present at the time of diagnosis in most patients and their frequency and severity increase as the disease progresses.

Worsening dyspnoea is a primary indicator of disease progression [40], including acute exacerbations, and should be evaluated at each visit. The optimal evaluation method has not been determined, but, in clinical practice, dyspnoea is usually assessed by a qualitative history or simple measures such as the modified Medical Research Council (mMRC) breathlessness scale. Dyspnoea is also a common manifestation of multiple comorbidities such as COPD, pulmonary hypertension and cardiovascular disease, and it is necessary to consider the worsening of dyspnoea in the context of other measures of disease progression. This allows us to determine whether dyspnoea worsening is due to IPF progression or not, and make decisions related to IPF pharmacotherapy, lung transplantation and/or palliative therapies.

Exercise capacity worsens as IPF progresses and increases morbidity and loss of independence. There is no established strategy for measuring physical function in IPF. Patients should be questioned about their ability to exercise at each visit. These questions focus on how far the patients can walk and whether they can climb stairs, with or without oxygen, as well as other questions related to the management of the patient at home.

Cough negatively affects quality of life in patients with IPF and tends to worsen as the disease progresses. In clinical practice, cough is evaluated qualitatively by frequency and severity.

In addition, we must also explore other symptoms such as sleep disturbance, depression, anxiety and pulmonary hypertension at each visit. Finally, we should assess the adverse effects derived from antifibrotic therapy [41].

### 8.2. Pulmonary Function Tests

Pulmonary function tests are an essential component of the disease control in IPF patients. The variables that are regularly monitored are FVC and DLco (both % predicted), strong predictors of mortality and key variables in timing for lung transplantation.

Spirometry, DLco and 6 MWT are usually performed at intervals of three to six months in patients with IPF, with more or less frequent testing depending on severity of symptoms and rate of worsening [13].

A decline in FVC or DLco of at least 10% over six to 12 months predicts an increased risk of mortality [34,36,42] and some studies suggest that decreases in 5% of FVC also portend a worse prognosis [43,44]. Those patients who present a decline in FVC and/or DLco should have this worsening interpreted in the context of other features of disease progression, such as symptoms and HRCT, given the inherent variability of the pulmonary function tests and the lack of specificity.

For patients with Combined Pulmonary Fibrosis and Emphysema (CPFE) or discordant changes in FVC and DLco, lung volumes by plethysmography may help clarify physiology. These patients have a relative preservation of spirometric values and a slower decrease in FVC compared to patients with isolated IPF [45,46], indicating the need to consider other functional parameters of disease progression in these patients.

Furthermore, 6 MWT is the most common method of measuring exercise capacity in patients with IPF. The most important variables obtained from the 6 MWT are the distance walked and extent of hypoxemia measured by pulse oximetry. A change in 6 MWD of approximately 30 meters is considered a clinically important change in IPF [15,47]. However, it is unknown what amount of change in oxygenation is clinically significant.

Both baseline and change in 6 MWD over six months are strong and independent predictors of mortality [18,48]. Lower values for the oxygen saturation nadir also indicate a worse prognosis [17]. These findings suggest that there may be a prognostic role to repeat the 6 MWT every six months, and particularly in patients with more severe disease.

The 6 MWT also provides data on when to start ambulatory oxygen supplementation. Patients with IPF who have exertional desaturation obtain symptomatic benefits from supplemental oxygen during exercise, although the long-term benefit of supplemental oxygen is not established in patients with isolated exertional hypoxemia and preservation of resting oxygen saturation [49].

Recently, more frequent monitoring of lung function by daily home spirometry is being explored as a way to identify acute exacerbations more rapidly as well as worsening of disease [50].

### 8.3. Oxygenation

Patients with advanced IPF often have hypoxemia at rest and require the administration of supplemental oxygen, which can be administered continuously, with ambulation, and at night as needed.

Development of worsening hypoxemia and increased oxygen requirements are common indicators of disease progression and increased mortality risk [9]. However, there is no established method to identify which patients require an assessment of resting, exertional, or nocturnal hipoxemia. Severe dyspnoea with ambulation, functional limitation secondary to dyspnoea, moderately or severely reduced DLco and the presence of pulmonary hypertension are common features to consider ambulatory oxygen administration.

Assessment of resting oxygenation by pulse oximetry is easily performed and often obtained in each clinical visit, particularly in patients with advanced disease.

Overnight oximetry is obtained in patients with clinical suspicion of nocturnal hypoxemia and/or sleep disorders. Given the absence of clear guidance on the frequency that patients should be evaluated, most patients are tested at baseline and when there is evidence of worsening disease with concern for nocturnal hypoxemia. The high prevalence of obstructive sleep apnea in patients with IPF indicates the need to consider nocturnal oximetry and polysomnography in all patients with IPF, even when there is a low suspicion of nocturnal hypoxemia [51,52].

An arterial blood gas is obtained in some patients as a complementary study of pulse oximetry in patients with borderline findings; however, this is a more invasive test and is often not necessary to demonstrate the presence of clinically significant hypoxemia. Respiratory acidosis is a rare finding in IPF and arterial blood gases are rarely obtained for the acid-base evaluation.

### 8.4. Chest Imaging

High-resolution computed tomography is essential for diagnosis of interstitial lung diseases, particularly in IPF. However, chest radiography has a limited role for routine monitoring of disease progression in patients with IPF, but it may be useful for the initial screen for potential aetiologies of acute respiratory worsening and may occasionally suggest other comorbidities.

High-resolution computed tomography, with or without a rule-out pulmonary embolism protocol, is an important test in the evaluation of an acute respiratory worsening (acute IPF exacerbation). Given the baseline abnormalities in IPF, comparison of HRCT findings during an episode of acute worsening of dyspnoea with a previous exam is essential.

The role of HRCT for monitoring IPF progression is less clear. In clinical practice, HRCTs are primarily assessed qualitatively to confirm or exclude significant disease progression, particularly in patients with discordant symptoms and physiology. Many clinicians repeat the HRCT every one or two years in patients with IPF, to identify any change in morphology, to clarify disease progression or to detect a new comorbidity (i.e., lung malignancy), even follow-up HRCT can identify a change in morphology that indicates the need to reconsider an initial diagnosis of IPF.

In addition to qualitative assessment, an experienced chest radiologist can visually score the severity of fibrosis on HRCT to obtain a total fibrosis score (percentage of affected lung) or calculate quantitatively using a computer algorithm to re-produce a fibrosis score. Previous studies have shown that both visual and computer-based scoring of fibrosis severity provide prognostic information beyond standard clinical and physiological variables [24,53,54,55]; however, these scores have been used mainly as research tools in specialized settings.

### 8.5. Laboratory Tests

No laboratory studies have been established as having a prognostic role in IPF. The laboratory testing is focused on characterization of intercurrent diseases, acute exacerbations and adverse effects of medication.

Serological evaluation for a connective tissue disease is recommended in the initial evaluation of IPF [2], including antinuclear antibodies, rheumatoid factor, and cyclic citrullinated peptide. Up to 10% of patients with a previous diagnosis of IPF can later develop a connective tissue disease [56], indicating that serological testing should be performed from time to time in IPF patients with features suggestive of a potential rheumatic disease. Conversely, up to 30% of IPF patients have positive autoimmune serology despite no other evidence of a rheumatic disease [57], indicating the poor specificity of autoimmune serologies for overt connective tissue disease in this population. It is not clear whether all patients with a diagnosis of IPF should have a repeated serologic evaluation on a regular basis after the initial evaluation.

Previous studies have suggested a possible prognostic role for molecular markers of lung damage (Table 2); however, these biomarkers do not have sufficient validation to support their clinical use in monitoring the progression of IPF.

## Figures and Tables

**Table 1:** Factors proved to be predictors of mortality.

Baseline	Follow-Up (6–12 Months)
Male gender; age >70 years	Grade of dyspnea increasing
Diagnosis delay	Exacerbations
Grade of dyspnea	Decline %FVC > 10%
Cardiovascular comorbidities	Decline %DLco > 15%
DLco ≤ 40%	Decline > 50 m at 6 MWT
SpO_2_ < 88% in 6 MWT	MCID variation in %FVC 2–6%
Distance 6 MWT < 250 m	MCID variation in 6 MWT 24–45 m
Fibrosis extension in HRCT	Fibrosis extension increasing in HRCT
Pulmonary hypertension	Complications
Biomarkers *	Biomarkers *
Increase of fibroblastic foci in surgical lung biopsy	
Multidimensional scales *	Longitudinal multidimensional scales *

* Waiting for reliable validation; Abbreviations: DLco: diffusion of carbon monoxide; FVC: Forced Vital Capacity; SpO_2_: Oxygen saturation by pulse oximetry; 6 MWT: 6 min walking test; HRCT: High-resolution computed tomography; MCID: minimal clinically important difference.

**Table 2:** Biomarkers related to idiopathic pulmonary fibrosis (IPF) worse prognostic.

**Serum**	KL-6 MUC-1 SP-A and SP-D	CCL18 CXCL13 S100A12 IL-8	MMP-7	LOXL2 ICAM-1 VCAM-1
**BAL**	MMP-9	Microbiome (Bacteroides, Fimicutes, Proteobacteria…)		
**Lung biopsy**	Integrin protein αvβ6	Shortening of telomeres		
**Genetics**	MUC5B gene polymorphism	Shortening of telomeres in white blood cells		

Abbreviations: BAL: bronchoalveolar lavage; KL-6: Krebs von den Lungen-6; MUC-1: mucin 1; SP-A: surfactant protein A; SP-D: surfactant protein D; MMP-9: matrix metalloproteinase 9; *MUC5B*: mucin 5B; CCL18: Chemokine (C-C motif) ligand 18; CXCL13: chemokine (C-X-C motif) ligand 13; S100A12: S100 calcium-binding protein A12; IL-8: interleukine-8; MMP-7: matrix metalloproteinase 7; LOXL2: lysyl oxidase-like-2; ICAM-1: intercellular adhesion molecule 1; VCAM-1: vascular cell adhesion molecule 1.

**Table 3:** Du Bois Prognostic Index.

Risk Factors	Score	Total Risk Score	Expected 1-Year Risk of Death
**Age**		0–4	<2%
≥70	8	8–14	2–5%
60–69	4	16–21	5–10%
<60	0	22–29	10–20%
**History of respiratory hospitalization**		30–33	20–30%
Yes	14	34–37	30–40%
No	0	38–40	40–50%
**% Predicted FVC**		41–43	50–60%
≤50	18	44–45	60–70%
51–65	13	47–49	70–80%
66–79	8	>50	>80%
≥80	0		
**24-Week change in % predicted FVC**			
≤−10	21		
−5 to −9.9	10		
>−4.9	0		

Modified from [36].

**Table 4:** Gender, Age, Physiology (GAP) Index.

Predictor		Points	
**Gender**			
Female		0	
Male		1	
**Age, years**			
≤60		0	
≤61		1	
≤62		2	
**Physiology**			
FVC, % predicted			
>75		0	
>76		1	
>77		2	
**DLco, % predicted**			
>55		0	
36–55		1	
≤35		2	
Cannot perform		3	
**Stage**	I	II	III
**Points**	0–3	4–5	6–8
**Mortality (%)**			
1-year	5.6	16.2	39.2
2-year	10.9	29.9	62.1
3-year	16.3	42.1	76.8

Modified from [35].

**Table 5:** Longitudinal GAP index.

Predictors	1-Year Mortality Score	2-Year Mortality Score
**Gender**		
Male	1	1
Female	0	0
**Age, years**		
>65	4	4
61–55	1	2
≤60	0	0
**Baseline FVC, % predicted**		
<50	15	12
50–75	12	9
≥75	0	0
**Baseline DLco, % predicted**		
Unable to perform	23	20
≤35	11	10
36–55	6	6
>56	0	0
**Δ FVC, % predicted**		
≤−10	12	10
−10 to −5	5	4
>−5	0	0
**History of respiratory hospitalization**		
Yes	14	13
No	0	0
Predicted Risk	1-Year Mortality Score	2-Year Mortality Score
<2%	0–10	-
2–5%	11–19	0–6
5–10%	20–26	7–13
10–20%	27–34	14–20
20–30%	35–38	21–25
30–40%	39–42	26–29
40–50%	43–45	30–32
50–60%	46–48	33–35
60–70%	49–51	36–37
70–80%	52–54	38–40
≥80%	55–69	41–60

Modified from [36].

**Table 6:** Risk stratificatiOn ScorE (ROSE score).

Low Risk (All three Criteria)	Intermediate Risk (One of the Following Criteria)	High Risk (All Three Criteria)
mMRC ≤ 3	mMRC > 3	mMRC > 3
6 MWD > 72% predicted	6 MWD ≤ 72% predicted	6 MWD ≤ 72% predicted
CPI ≤ 41	CPI > 41	CPI > 41

Abbreviations: mMRC: Modified Medical Research Council Breathlessness Scale; 6 MWD: six minutes walking test distance; CPI: Composite Physiological Index. Modified from [37].

**Table 7:** Main examinations during follow-up period.

Early Disease	Advanced Disease
Every 3–6 Months	Every 3 Months or Less
Symptoms evaluation	Symptoms evaluation
Hospitalizations	Hospitalizations
FVC, DLco	FVC, DLco
6 MWT	6 MWT
Pulse oximetry	Pulse oximetry
Optional: HRCT *, ecocardiography	Optional: HRCT *, ecocardiography

* HRCT is indicated to evaluate morphology changes, clarifying disease progression or diagnose new comorbidities (lung cancer).
